# A mosaic of microclimates: biodiversity outcomes and wildlife habitat potential in large‐scale solar facilities

**DOI:** 10.1002/brv.70171

**Published:** 2026-04-12

**Authors:** Tom Armstrong, Caleb L. Loughran, Joshua R. Ennen, Kristoffer H. Wild, Eric J. Nordberg

**Affiliations:** ^1^ Ecosystem Management, School of Environmental and Rural Science, University of New England Armidale NSW 2351 Australia; ^2^ Department of Natural Sciences Western New Mexico University 1000 W. College Ave Silver City NM 88061 USA; ^3^ Department of Biology University of New Mexico MSC03‐2020 Albuquerque NM 87131‐0001 USA; ^4^ Renewable Energy Wildlife Institute 700 12th Street, NW Suite 700 Washington DC 20005 USA; ^5^ School of BioSciences, The University of Melbourne Melbourne VIC 3010 Australia

**Keywords:** biodiversity, environmental conditions, habitat suitability, moisture, renewable energy, temperature

## Abstract

The rapid global expansion of photovoltaic (PV) solar facilities, now comprising nearly 80% of the recent and projected growth of renewable electricity, represents one of the most significant land‐use changes of the 21st century. While PV facilities are critical for decarbonising energy systems, their large spatial footprint and infrastructure fundamentally alter local abiotic conditions, with potential cascading effects on ecosystems and biodiversity. This review synthesises findings from 33 peer‐reviewed publications across diverse climates and ecosystems, examining how large‐scale solar facilities modify microclimates. Key environmental variables affected include temperature, humidity, wind speed, soil moisture, albedo, and photosynthetically active radiation. Generally, PV arrays create spatially heterogeneous microclimates: air temperatures are often elevated above panels, while soils beneath tend to be cooler, more stable, and retain more moisture due to shading. However, these patterns are not universal. Generalisations are limited by small sample sizes, variation in sensor placement, and differences in study design, so results may not apply to all PV facilities or biomes. The effects of PV arrays on microclimate are likely context dependent, varying with local climate, facility design, and ground‐cover management. This review highlights the need for standardised microclimate research and reporting within solar facilities to understand environmental variability better across sites and regions. Integrating microclimate studies with ecological modelling and habitat suitability assessments will help researchers and land managers determine how species interact with environments within and around PV facilities. Leveraging such insights can guide facility design, inform habitat restoration, and support the sustainable development of solar energy.

## I. INTRODUCTION

The global energy transition towards renewable sources has led to unprecedented growth in the development of photovoltaic (PV) solar facilities. As of 2024, PV solar facilities, both large and small scale, account for nearly 80% of the past and forecasted growth of renewable electricity between 2024 and 2030 (International Energy Agency (IEA), 2024). The larger installations often occupy hundreds to thousands of hectares of land due to their low land‐use efficiency (i.e. a large amount of land is required per unit of energy production: 12.30–16.97 km^2^/TWhr; Trainor, McDonald & Fargione, 2016). This rapid expansion and scale of development represents one of the fastest land‐use changes in history, raising ecological and environmental concerns at various spatial scales, from local to landscape (Turney & Fthenakis, 2011; Agha *et al*., 2020; Blaydes *et al*., 2025).

Among these concerns, the modification of localised abiotic conditions (i.e. microclimates) is emerging as a key, yet underexplored, dimension of solar development effects. Microclimates are local areas where the balance of energy (i.e. heat transfer), comprising solar radiation, convection and conduction (i.e. heat gain and loss), and the effects of wind and humidity, are maintained according to the principles of the first law of thermodynamics (Porter & Gates, 1969; Porter *et al*., 1973; Kearney & Porter, 2009). In an ecological context, microclimates are the localised suite of physical conditions (e.g. temperature, humidity, solar radiation, soil moisture, and wind speed) that limit the persistence of plant and animal species in a given area. For many organisms, access to a mosaic of microclimates enables them to select conditions that permit physiological optima by maintaining optimal body temperature, avoiding critical thermal limits, mitigating evaporative water loss, sustaining stable metabolism, and supporting regular activity (Kearney & Porter, 2009; Scheffers *et al*., 2014). Thus, microclimates play a pivotal role in shaping species responses, community dynamics, and ecosystem functions.

Despite the rapid proliferation of solar installations worldwide, the influence of solar facilities on abiotic conditions, both onsite and offsite, remains poorly understood and often contradictory. PV solar facilities modify surface albedo, causing more absorption of incident radiation due to the panels' dark colour and potentially the photovoltaic heat island (PVHI) effect (e.g. the release of heat from solar panels) above and surrounding PV solar facilities (Millstein & Menon, 2011; Barron‐Gafford *et al*., 2016). In one example of the PVHI, Broadbent *et al*. (2019) reported air temperature increases of 3–4 °C during the day and 1–2 °C at night relative to the adjacent open desert in Arizona, USA. The elevated panels also limit solar radiation from reaching the ground, creating complex and dynamic shade regimes, and influence wind speed (Noor & Reeza, 2022; Choi *et al*., 2023; Zheng *et al*., 2023; Sturchio, Kannenberg & Knapp, 2024a). These microclimate mosaics, characterised by heating (Millstein & Menon, 2011; Barron‐Gafford *et al*., 2016) or cooling effects (Guoqing *et al*., 2021; Xu *et al*., 2024), along with the redistribution of soil moisture, and the mitigation of extreme temperatures (Choi *et al*., 2023), appear to be dependent on geographic characteristics, onsite groundcover composition, and design and methodology of facility installation. These microclimatic shifts fundamentally alter the abiotic features to which organisms respond. However, the direction and magnitude of these shifts likely vary across biomes, regions, and installation designs, thus making it challenging to provide general predictions about the ecological effects of the PV solar‐induced microclimates.

The direct effects of PV solar facilities on species include habitat loss and fragmentation, changes in animal behaviour (e.g. attraction, avoidance, or thermoregulation), shifts in population dynamics (e.g. fatalities, altered dispersal, and reproduction), and alterations in community composition [e.g. biodiversity and invasive species (Lovich & Ennen, 2011; Karban *et al*., 2024)]. Yet, the direct and indirect effects induced by microclimate shifts are less well understood and may either ameliorate or exacerbate some of the direct effects, such as onsite behaviour and demography. These shifts may also alter the quality, availability, and structural diversity of species‐specific habitats necessary to support certain life‐history functions, but also influence soil–plant interactions, which are the basis of lower trophic levels, and broader ecosystem‐level processes [e.g. nutrient cycling, soil conditions, and water cycling (Grodsky & Hernandez, 2020; Heredia‐Velásquez *et al*., 2023; Karban *et al*., 2025; Siggers *et al*., 2025)]. By modifying the average microclimate conditions and the extent to which they vary, PV solar facilities can fundamentally reshape the biophysical niche that organisms occupy. The reshaped microclimate conditions are important for animals, particularly ectotherms, that rely on thermal heterogeneity to regulate physiological processes (Huey, 1991), such that small‐scale spatial variability expands the usable habitat space under extreme conditions (Paaijmans *et al*., 2013; Logan, Fernandez & Calsbeek, 2015), which can be mitigated by PV solar facilities (Choi *et al*., 2023; Sturchio & Knapp, 2023).

While it is well established that microclimates have significant effects on how organisms interact with their environment, the microclimatic shifts induced by PV solar facilities, and their consequences for ecological processes, remain poorly understood. This knowledge gap is partly due to the broad range of microclimatic effects observed across global biomes and the variation in PV installation design and infrastructure (e.g. fixed or single‐axis racking systems). Also, a lack of standardised measurements, sensor placements, and reporting conventions makes cross‐site comparisons and generalisations difficult and therefore, makes it harder to predict effects on species, populations, and communities. While a recent review examined solar‐induced microclimates in agrivoltaic systems, mainly with an emphasis on crop production and in agricultural systems (Omer *et al*., 2025), it did not systematically account for how the broader climatic context influences microclimate responses, nor did it include wind speed as a key factor, despite its importance for shaping microclimates for many species and ecosystems. Agrivoltaic systems integrate solar energy production with agricultural activities such as cropping or livestock grazing, whereas ecovoltaic (or conservoltaic) systems prioritise ecological principles, biodiversity conservation, and habitat restoration alongside energy generation (Sturchio & Knapp, 2023; Nordberg & Schwarzkopf, 2023). Both approaches represent dual land‐use strategies that may benefit from, or be constrained by, the microclimatic conditions created by PV infrastructure. Yet the ecological implications of these microclimate shifts remain underexplored for native organisms in both system types.

Here, we address these gaps by reviewing the ecological effects of PV‐induced microclimate modification and its role in shaping habitat suitability for wildlife, their habitats, and ecosystem processes. We conducted a systematic literature review on PV solar‐induced abiotic conditions and their local and regional outcomes. Although ecological responses can arise due to construction‐related disturbance, this review focuses on microclimate shifts as they pertain to established PV installations. Our review synthesises the current understanding of microclimates within PV solar facilities, identifying research needs, and providing a framework for future investigations. In this review, we ask: *are the ways in which PV solar arrays shape microclimates consistent across studies* and *which variables are most consistently affected?* We briefly discuss the implications of PV solar‐induced microclimates on ecological processes and functions. We highlight the need for standardised characterisation of microclimates and provide recommendations for future directions.

## II. METHODS

### (1) Literature search

To assess how solar energy infrastructure influences microclimates, we systematically searched for peer‐reviewed studies examining the microclimatic conditions within and around PV solar facilities. On 20 March 2025, we conducted a systematic literature search in *Web of Science* (core collection) and *PRIMO*. We did not apply a timespan limit. The search included terms related to solar energy infrastructure (‘solar farm’, ‘solar park’, ‘photovoltaic solar’, ‘PV solar’, and ‘utility‐scale solar’) combined with terms related to microclimate (AND ‘microclimate’ OR ‘micro‐climate’). To capture relevant environmental and climatic factors, additional key words included: OR ‘cool island’, ‘heat island’, ‘abiotic’, ‘albedo’, ‘reflectivity’, ‘radiation’, ‘thermal’, ‘humidity’, ‘moisture’, ‘soil moisture’, ‘precipitation’, ‘infiltration’, ‘runoff’, ‘shade’, ‘surface temperature’, ‘temperature’, ‘wind’, and ‘soil’. By strategically combining these search terms, we aimed to identify literature focused on the effects of large‐scale PV solar energy developments on local climate conditions, specifically temperature regulation, moisture dynamics, and surface energy balance. Our stringent search criteria, which required the inclusion of ‘microclimate’ or ‘micro‐climate’, intentionally excluded studies centred on PV engineering, technical construction, or other aspects of solar energy not directly related to microclimate effects. To ensure a comprehensive review, we also examined the reference lists of relevant journal articles and reviews, incorporating additional literature that may have been overlooked in the primary search. This approach strengthened the breadth and depth of our analysis.

**Fig. 1:**
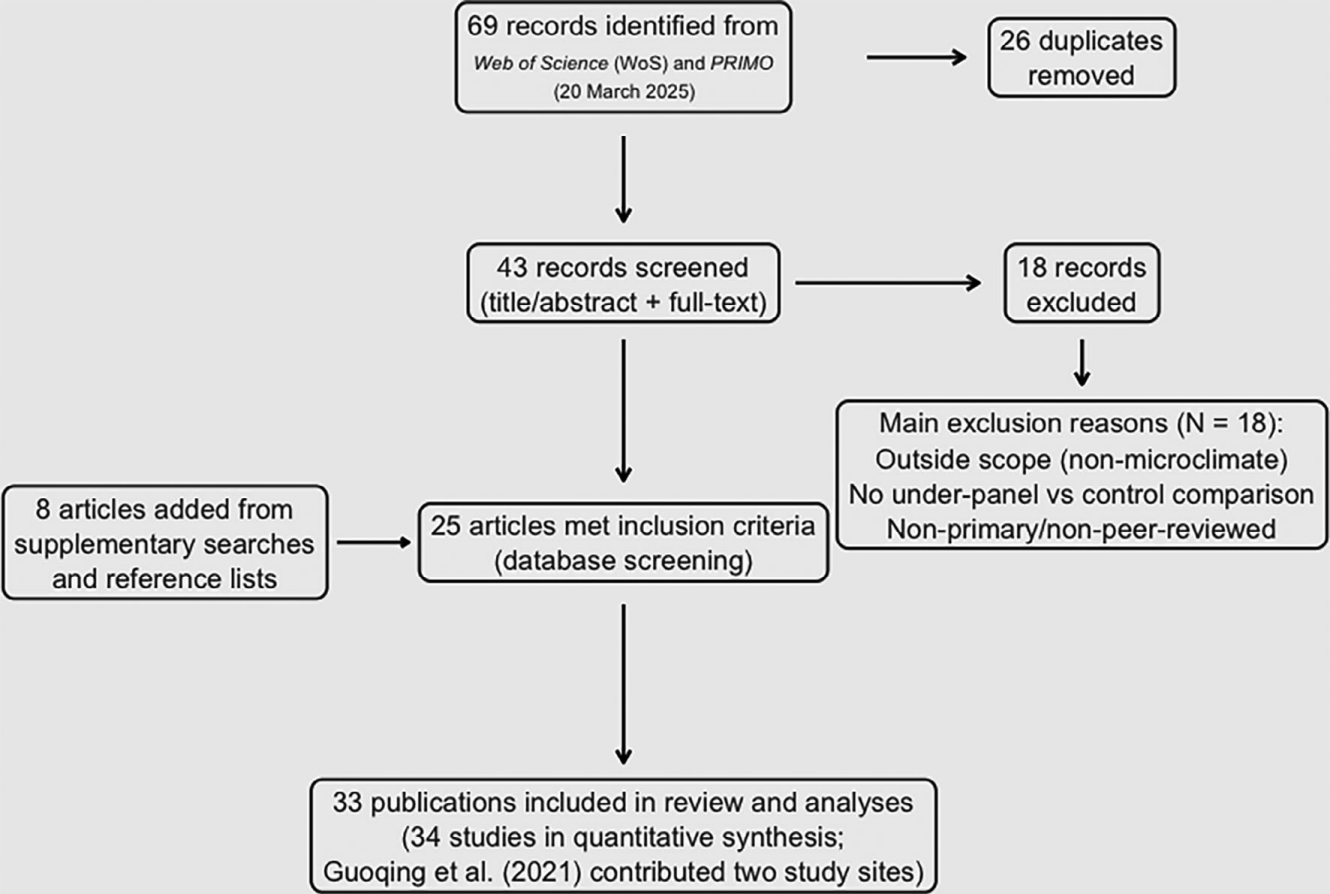
Flow chart illustrating the systematic literature search and screening process used in this review. One publication (Guoqing *et al*., 2021) reported two independent study sites and was treated as two separate studies in the quantitative synthesis (33 publications; 34 studies).

After removing duplicates (*N* = 26) from the initial search results, the remaining journal articles (*N* = 43) were screened using predefined inclusion and exclusion criteria, beginning with title and abstract screening and followed by full‐text review (Fig. 1). A total of 18 records were excluded because they did not meet our selection criteria: (*i*) they did not fit the scope of our study (not related to microclimate conditions on solar farms); (*ii*) there were no treatment *versus* control comparisons; or (*iii*) the record was published only as ‘grey literature’ (not a primary/peer‐reviewed study). One publication (Guoqing *et al*., 2021) reported results from two independent study sites with separate data sets and was therefore treated as two distinct studies in the quantitative synthesis. Consequently, the final data set comprised 34 studies derived from 33 publications. We extracted data from each study, including facility location, climate, structural design parameters (e.g. panel height, sensor location, sensor height and depth, mounting system type, agrivoltaic system type, distance between panel rows, and ecosystem type), and environmental parameters [e.g. air and soil temperature, humidity, soil moisture, wind speed, albedo, photosynthetically active radiation (PAR), soil stability, and soil water infiltration] measured under solar panels compared to between panel rows or control areas. See online Supporting Information Table S1 for included studies and extracted data. Unless otherwise specified, reported microclimate variations refer to differences relative to a control. Abbreviations and definitions of extracted environmental and climatic variables are provided in Table 1.

**Table 1:** Glossary of variables and terms extracted and discussed herein.

Variable	Definition
Air Temp (°C)	Air temperature measured at varying heights above ground level.
Soil moisture (%)	Volume or percentage of water retained within the soil profile.
Soil temp (°C)	Temperature recorded at specific depths within the soil.
Humidity (%)	Concentration of water vapour present in the air.
PAR (μmol m^−2^ s^−1^)	Photosynthetically active radiation (400–700 nm) available for plant growth.
Surface radiation (W m^−2^)	Net balance of incoming and outgoing radiation at the ground surface.
Wind (m s^−1^)	Speed and direction of air movement near the surface
Albedo	Proportion of incoming solar radiation reflected by the surface.
Soil infiltration	Rate at which water is absorbed into the soil.
Soil stability	Resistance of soil to erosion, compaction, and structural degradation.
Land surface temperature	Temperature of the land surface measured *via* remote sensing.

## III. RESULTS

### (1) Geographic trends and land‐use

**Fig. 2:**
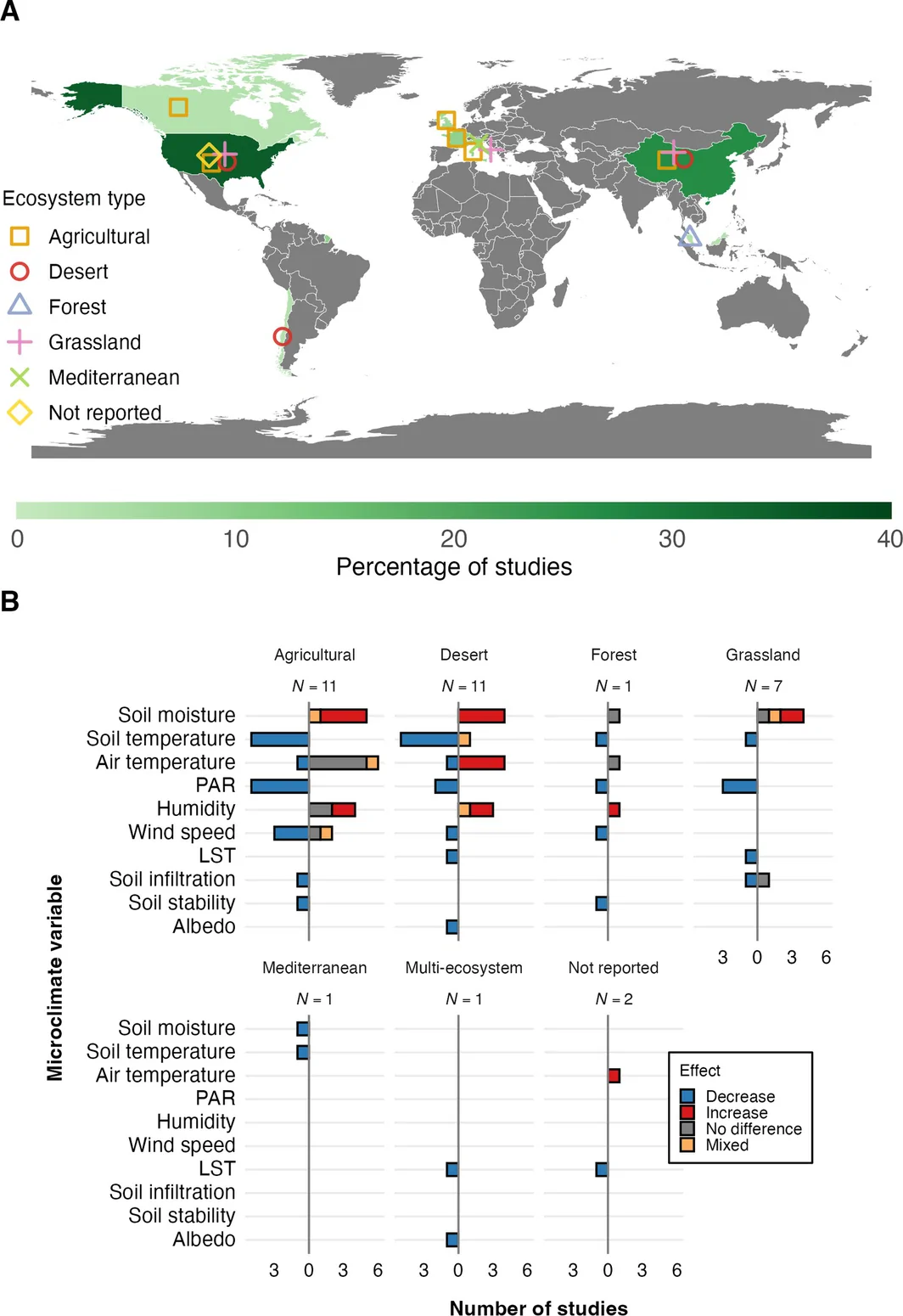
Results of the systematic review assessing the effects of solar energy infrastructure on microclimate. (A) Global distribution of studies by ecosystem type. Country shading indicates the percentage of all studies conducted in each country, and symbols indicate the ecosystem types reported. Where site coordinates were unavailable, points were placed at country centroids. Global studies were included in summary totals but are not shown on the map. (B) Summary of microclimate variables measured across ecosystem types. Facet labels show the number of studies per ecosystem type (*N*). Bar colours indicate the direction of effects under solar panels relative to control areas (decrease, increase, no difference, mixed). LST, land surface temperature; PAR, photosynthetically active radiation.

Of the 34 studies reviewed, the USA contributed the largest proportion (35.3%; 12/34), followed by China (26.5%; 9/34) and both France and Italy (8.8%; 3/34; Fig. 2A, Table S1). Other countries, such as the UK, Chile, Canada, and Malaysia, as well as global‐scale studies, each accounted for less than 5.8% of the total. Regarding ecosystem type, solar facilities developed on agricultural and desert ecosystems were studied equally (32.4%; 11/34), grasslands (20.6%; 7/34), Mediterranean systems (2.9%; 1/34), and forests (2.9%; 1/34). The ecosystem type was not reported in 5.9% (2/34) of studies. Solar–grazing activities (e.g. sheep grazing) were reported in 14.7% of studies (5/34), while 20.6% (7/34) indicated no grazing activity, and 64.7% (22/34) did not specify. Cropping under solar panels was reported in 8.8% of studies (3/34), including one modelling study. Only one study characterised species niche spaces or habitat suitability based on abiotic parameters (Suuronen *et al*., 2017).

### (2) Trends in solar infrastructure design, study types, and sensor placement

Of the 34 studies reviewed, 70.6% (24/34) were observational, 11.8% (4/34) used modelling simulations,11.8% (4/34) used remote sensing with Earth observation satellites, and 5.9% (2/34) combined either observational field methods with modelling or remote sensing with modelling. Sensor placement was reported in 63% of studies reporting a microclimate measurement (17/27). Specifically, sensor‐position details appeared in 85.7% of air temperature studies (12/14), 71.4% of soil temperature studies (10/14), 87.5% of relative humidity studies (7/8), 60.0% of soil moisture studies (9/15), 0% of PAR studies (0/11), and 57.1% of wind studies (4/7).

The electrical capacity of solar installations ranged from 1 to 3,182 MW. Excluding the outlier (3,182 MW), the mean ± SD capacity was 110.45 ± 256.9 MW. The mounting design in 55.9% of the studies (19/34) was fixed‐tilt, while 17.6% (6/34) were single‐axis tracking systems, and 23.5% of the studies (8/34) did not specify the mounting system. One study directly compared microclimate variations between the two mounting types (Suuronen *et al*., 2017). The lowest point between the PV panel and ground ranged from 0.4 to 4 m, with a mean ± SD of 1.32 ± 1.05 m. The interspace between panel arrays ranged from 1 to 11 m, with a mean ± SD of 4.97 ± 2.58 m. Sensor placement varied across studies. The mean ± SD height of air temperature sensors was 1.43 ± 0.87 m and ranged from 0.1 to 2.7 m. Soil temperature sensors had a sub‐surface depth range of 0.02–0.4 m with a mean ± SD of 0.13 ± 0.11 m. Humidity sensors had a height range of 0.1–2.7 m with a mean ± SD of 1.32 ± 0.86 m. Soil moisture sensors had a sub‐surface depth range of 0–0.4 m with a mean ± SD of 0.15 ± 0.11 m and wind sensor heights ranged from 0.5 to 2.7 m, with a mean ± SD of 1.36 ± 0.78 m. There was no consistency in sensor placement, either in terms of height/depth or location within the solar facility, when measuring the same microclimate variables across different studies. Additionally, 23.5% (8/34) of the studies did not record data from outside the solar facility.

### (3) Microclimate variables

#### (a) Air temperature and land surface temperature (LST)

Air temperature was measured in 41.2% of studies (14/34), with reported effects of solar facilities on air temperature showing no significant difference in 42.9% (6/14), increases in 35.7% (5/14), and decreases in 14.3% (2/14) of studies when comparing areas beneath panels to control areas. One study reported mixed results. Land surface temperature (LST) was measured in 11.8% of studies (4/34), all of which reported a decrease within solar farm areas relative to surrounding areas. The air temperature responses varied according to within‐ and among‐site‐context variables, but no clear pattern emerged due to low sample sizes and the failure to report key variables, including the ecosystem, system design, sensor height, and other facility contexts. In the Sonoran Desert of Arizona, USA, daytime exposed (i.e. not in panel shade) air temperatures measured at 1.5 m above ground using HOBO Pro v2 sensors were, on average, 1.38 °C warmer within the solar facility between panel rows (Broadbent *et al*., 2019). By contrast, night temperatures showed no significant difference (Broadbent *et al*., 2019). A nearby study using probes at 2.5 m above ground recorded nighttime warming of 3–4 °C on the solar facility, particularly during spring and summer (March – August) (Barron‐Gafford *et al*., 2016). Additionally, these increased air temperatures can be detected over 300 m outside the PV solar facility, with consequences to plants (e.g. water and heat stress; Devitt *et al*., 2022), and potentially non‐volant wildlife (Lovich & Ennen, 2011; DeGregorio *et al*., 2015). Remote‐sensing analyses revealed contrasting patterns at ground level: Zhang & Xu (2020) documented LST cooling in arid and semi‐arid solar installations across China, India, and the USA, while Guoqing *et al*. (2021) reported surface cooling effects extending 730 m beyond PV facility boundaries in desert and grassland regions of China and the USA, with reductions up to 2.3 °C in adjacent buffer zones. In Chile's Atacama Desert, shaded sensors placed 10 cm above ground recorded cooler air beneath fixed‐mount PV panels compared to exposed areas (Suuronen *et al*., 2017). However, the same study found that tracking systems produced weaker cooling effects, with air temperatures under the panels more closely resembling sunlit areas due to reduced shading duration. In temperate agricultural grasslands of the UK, sensors 50 cm from the ground exhibited reduced diurnal variability beneath PV panels, with cooler daytime temperatures (up to 5.2 °C in summer) and warmer nighttime temperatures relative to control areas (Armstrong, Ostle & Whitaker, 2016). Air temperatures measured at 50 cm above ground showed no significant difference between PV‐covered and open areas in tropical Malaysia, likely due to the consistently warm and humid conditions (Noor & Reeza, 2022). Computational fluid dynamics models, validated against weather station data collected 2.5 m above ground, showed air temperature cooling by up to 10 °C underneath panels when panels were mounted at 4 m above soybeans, driven by evapotranspiration, high ground albedo, and enhanced convective airflow (Williams *et al*., 2023).

#### (b) Humidity

Relative humidity was reported in 23.5% of studies (8/34), with reported solar facility effects showing increases in 62.5% (5/8), no significant difference in 25.0% (2/8), and mixed results in 12.5% (1/8) of studies. Desert, agricultural, and forested ecosystems showed increased humidity beneath panels, and relative humidity was consistently higher in shaded micro‐patches, particularly during early‐morning and night‐time hours (Vervloesem *et al*., 2022). In tropical Malaysia, humidity reached 65% beneath panels, measured 50 cm above ground, compared to 53–63% in exposed areas, likely due to reduced evapotranspiration (Noor & Reeza, 2022). In desert environments, humidity was higher under panels in autumn and winter (September–February) but lower in spring and summer (March–August), with reduced diurnal variability indicating a buffering effect in the Gobi Desert (Zheng *et al*., 2023). In the Atacama Desert, fixed panels increased relative humidity more than tracking systems, likely due to more consistent shading by the fixed system (Suuronen *et al*., 2017).

#### (c) Wind speed

Wind speed was reported in 20.6% of studies (7/34). Of these, 71.4% (5/7) reported decreases in wind speed, 14.3% (1/7) reported mixed effects across sensor heights, and 14.3% (1/7) reported no significant differences. Most wind speed studies were conducted in agricultural systems (71.4%, 5/7), with 71.4% (5/7) using external control sites and 28.6% (2/7) using internal controls. Four of the seven studies (57.1%) reported sensor heights, which ranged from 0.5 to 2.7 m (mean ± SD = 1.36 ± 0.78 m). Both fixed‐tilt and tracking systems reduced wind speeds beneath panels by 30–56%, primarily due to physical obstruction and, in some cases, increased vegetation roughness (Noor & Reeza, 2022; Choi *et al*., 2023; Zheng *et al*., 2023). Wind suppression was greatest in central, low‐lying zones of the solar installation studied in tropical Malaysia. A fixed‐tilt solar installation on agricultural land recorded wind speeds 1.3–1.85 times higher in open areas than under the panels (Li *et al*., 2025).

#### (d) Soil temperature

Soil temperature was reported in 41.2% of studies (14/34). Of these, 92.9% (13/14) reported decreases in soil temperature, and 7.14% (1/14) reported mixed effects across sensor depths. Soil temperature was measured most often in agricultural (35.7%, 5/14) and desert (42.9%, 6/14) ecosystems. The majority used external control sites (71.4%, 10/14), while 28.6% (4/14) used internal controls. In tropical Malaysia, soil temperatures at the edges of a PV site (i.e. full‐sun plots) were significantly higher than those beneath or between fixed‐tilt panels, suggesting a cooling effect from panel shade (Noor & Reeza, 2022).

In agricultural California, exposed soil measured at a 10 cm depth beneath a single‐axis tracking system was up to 4.9 °C warmer than soil at the same depth located within shaded areas under the panels of a single‐axis tracking system (Li *et al*., 2025). In cold‐arid grasslands of northeastern China, soil temperature was lower in PV array zones than in adjacent ambient grassland, reflecting strong shading effects (Zhang *et al*., 2023). In arid southern Arizona, soil beneath single‐axis tracking panels, measured at a depth of 2 to 6 cm, was up to 9.8 °C cooler than nearby exposed bare ground. The maximum seasonal differences reached 14.8 °C in spring, primarily due to shading and reduced ground heat flux (Broadbent *et al*., 2019). In desert China, vertical profiles showed that soil temperature under both fixed‐tilt and single‐axis panels decreased with increasing depth in spring and summer (March–August) but increased with depth in autumn and winter (September–February). Fixed‐tilt panels cooled the 0–0.4 m soil by 3.3–4.0 °C in summer (and also lowered it in spring and autumn) while warming it by 1.5–2.3 °C in winter, whereas single‐axis panels produced cooling of 1.5–1.9 °C in summer (and cooling in spring and autumn) and a slight winter warming of 0.1–0.6 °C (Yue *et al*., 2021). In agricultural France, spring and summer (June–August) soil temperatures at 7 cm depth and beneath fixed panels were reduced by approximately 5 °C compared to inter‐row areas (Lambert *et al*., 2022a). Across all studies, known sensor depths ranged from 2 to 40 cm, with most studies focusing on the upper 10 cm (8/9, 88.9%) to capture near‐surface thermal dynamics relevant for vegetation and microbial processes.

#### (e) Soil moisture

Soil moisture was reported in 44.1% (15/34) of studies, with most (66.7%; 10/15) reporting higher soil moisture levels underneath the panels. Soil moisture outcomes are more nuanced due to variation in infrastructure and racking system. Single‐panel systems will redistribute soil moisture towards the edges, creating a dripline, whereas double‐panelled systems (i.e. two panels in portrait, see Fig. 1 of Wu *et al*., 2022) will redistribute soil moisture similarly but also have a drip line underneath the panel array due to the gap between panels (Choi *et al*., 2023). In desert ecosystems, all studies that measured soil moisture (4/11) reported higher levels beneath PV panels due to reduced evaporation and altered rainfall distribution. For example, Liu *et al*. (2022) observed increased soil moisture from 6.2% to 11.8% over 6 years in arid China, while Wu *et al*. (2022) modelled soil moisture increases of 59.8–113.6% in shaded zones. Yue *et al*. (2021) and Suuronen *et al*. (2017) similarly reported moisture increase under panels from shading and runoff concentration. In semi‐arid systems, soil moisture is characteristically heterogeneous rather than uniformly wetter or drier beneath PV arrays. In semi‐arid grasslands, both single‐event and seasonal observations show that moisture increases are often greatest along panel edges or driplines where runoff and throughfall accumulate, while other sub‐panel microsites remain comparatively dry (Choi *et al*., 2020; Sturchio *et al*., 2024b). At some agrivoltaic cropland sites, this same fine‐scale heterogeneity occurs but is accompanied by a broader decline in soil water‐holding capacity or winter soil moisture beneath panels compared with adjacent rows (Moscatelli *et al*., 2022; Li *et al*., 2025).

#### (f) Albedo

Albedo was rarely measured in agrivoltaic studies, with only 5.88% (2/34) reporting on surface reflectivity changes. Both studies documented decreases in albedo beneath solar installations. Broadbent *et al*. (2019) applied field‐based energy balance modelling in an Arizona desert and reported a decrease in albedo from 0.31 to 0.20, which contributed to local warming measured at 1.5 m above ground. Xu *et al*. (2024) analysed MODIS satellite data from 116 global solar sites across barren, cropland, and grassland ecosystems, finding a mean ± SD albedo decline of 0.016 ± 0.009, with the greatest reductions observed in desert regions.

#### (g) Photosynthetically active radiation (PAR)

PAR was measured in 32.4% of studies (11/34). All 11 studies reported decreases in PAR beneath solar panels. PAR was measured most often in agricultural systems (45.5%, 5/11), followed by grasslands (27.3%, 3/11), deserts (18.2%, 2/11), and forests (9.1%, 1/11). In a desert ecosystem in China, Li *et al*. (2023) observed an 87% reduction in PAR beneath panels, from approximately 745 to 98 μmol m^−2^ s^−1^. They identified this decline as a key driver of shifts in plant and microbial communities. On agricultural land, Li *et al*. (2025) reported that morning, afternoon, and full‐day shading by single‐axis tracking PV systems reduced PAR by 9–89% relative to unshaded areas. Similarly, Barron‐Gafford *et al*. (2016) found consistently lower PAR under PV arrays in a dryland agrivoltaic system, with associated effects on microclimate and plant physiological responses. Differences in racking height help explain this variation: taller 1.8–2.4 m tracking systems allow ~30–70% of full‐sun PPFD (photosynthetic photon flux density) to reach the ground (Sturchio *et al*., 2024b), whereas lower 1.37 m racking produces much larger declines, with PAR beneath panels dropping to ~89 μmol m^−2^ s^−1^ compared to 842 μmol m^−2^ s^−1^ in unshaded areas (Li *et al*., 2025).

#### (h) Soil stability

Soil stability was assessed in 5.9% (2/34) of studies, both of which reported declines within solar installations compared to controls. At the UiTM Solar Park in a tropical environment in Malaysia, sandy soils and sparse vegetation contributed to surface erosion during heavy rainfall (Noor & Reeza, 2022). In an agricultural ecosystem in southern France, Lambert *et al*. (2022a) found that soil aggregate stability, measured by mean weight diameter, was 1.5 times lower in solar facility soils than in surrounding pinewood and shrubland. Construction activities, such as soil levelling and vegetation removal, were identified as key drivers of reduced soil structure and increased erosion risk.

#### (i) Soil water infiltration

Soil infiltration was assessed in 8.8% (3/34) of studies. Two found decreased infiltration within solar facilities compared to external controls, while the third reported no significant difference. In temperate agricultural systems in England and Wales, Carvalho *et al*. (2025) observed that higher soil compaction and lower particulate organic matter beneath solar panels likely impaired infiltration over time. Similarly, modelling work in grassland ecosystems suggested that although short‐term runoff changes were minimal, long‐term vegetation loss could reduce surface roughness and increase runoff by up to 35%, implying declining infiltration capacity if ground cover is not maintained (Gullotta *et al*., 2023). By contrast, Choi *et al*. (2020) found no significant difference in infiltration rates between a revegetated solar PV site and adjacent reference grassland in Colorado, USA. However, within the PV site, infiltration was significantly higher directly beneath panels than at dripline or interspace locations, likely due to reduced soil compaction from limited maintenance activity (i.e. vehicle traffic and mowing) underneath panels.

## IV. DISCUSSION

### (1) Microclimate shifts

**Fig. 3:**
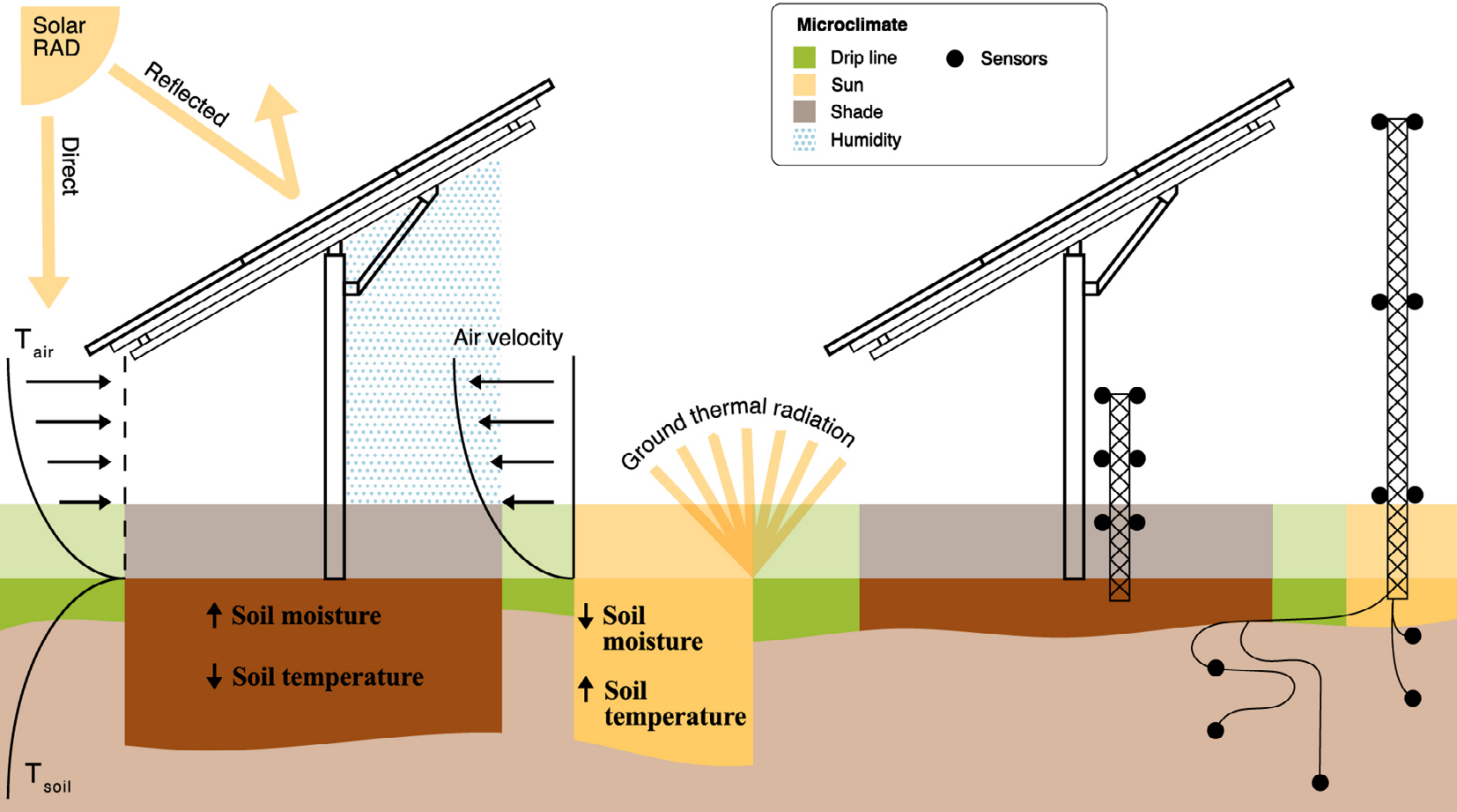
Conceptual diagram of microclimate variation and environmental monitoring beneath photovoltaic (PV) solar panels. The figure illustrates how solar panels alter microclimatic conditions through shading, redirected solar radiation (RAD), changes in air velocity, and ground thermal radiation. These modifications create spatial heterogeneity in temperature (*T*_air_, *T*_soil_), humidity, and soil conditions beneath and between panels. Beneath shaded zones (e.g. drip line), soil moisture tends to increase, and soil temperature decreases. Note that soil moisture redistribution is dependent on infrastructure and facility design (see text for explanation). Exposed zones show the opposite trend. Sensors for air and soil temperature, wind speed, and humidity placed across microhabitat zones – sun, shade, and drip line – are needed to capture these fine‐scale differences. This spatial variation may influence animal behaviour, particularly in ectothermic species, and affect broader ecosystem processes.

Our systematic review of 34 studies revealed considerable variation in how the effects of PV solar panels alter local microclimates, which may alleviate or exacerbate the biophysical challenges that organisms face within a given environment. This variation may be associated with inconsistencies in reporting context‐specific variables (e.g. climate, sensor placement, and racking system) that might influence microclimate outcomes. We found that 92.9% of studies (13/14) reported lower soil temperatures beneath panels, 66.7% (10/15) noted increased soil moisture, and 57.1% (8/14) recorded air temperature changes, often with warming effects above or between arrays (Fig. 3). This could raise air temperatures around facilities, create cooler shaded areas beneath panels, block near‐surface wind (reducing convective heat loss), create insulating effects under panels, and change soil moisture retention (Fig. 3). The resulting shifts in above‐surface, at‐surface, and sub‐surface microclimates could influence individual organisms that may inhabit solar facilities, as well as population‐level (Tanner *et al*., 2020; Hernandez *et al*., 2020) and community‐level responses (Suuronen *et al*., 2017; Karban *et al*., 2025). For example, in studies from desert, temperate, and tropical ecosystems, PV installations altered heat transfer dynamics by blocking radiation and creating shaded zones below panels, or amplifying radiation reflection above panels. Air temperature increases of up to 4 °C were observed above panels in Arizona (Barron‐Gafford *et al*., 2016), whereas soil temperatures were reduced by 5–14.8 °C in shaded zones across both arid and agricultural regions (e.g. Broadbent *et al*., 2019; Lambert *et al*., 2022a, 2022b). These changes often coincided with lower wind speeds (30–56% reduction) and redistributed soil moisture, typically increasing in arid systems and becoming more heterogeneous in agricultural settings.

Although we found that PV solar arrays are likely to create spatially heterogeneous microclimates, the above generalisations are limited by sample size and variation in sensor placement, and thus, may not apply to all PV solar facilities across all biomes. These effects appear to be highly context dependent, varying along the axes of: (*i*) local climate; (*ii*) facility design; and (*iii*) groundcover type (e.g. native plants, turfgrasses, or a combination of both) management (e.g. mechanical mowing and/or grazing), with the ability to capture this variation hinging on sensor design and placement. Below, we discuss how each facet of the microclimate structure may be affected by solar panels and the inconsistencies in sensor placement and reporting of context‐dependent factors (e.g. racking system and biome).

### (2) Integrating microclimate shifts into a unified framework

Microclimate variables are often recorded independently rather than as interacting components, yet the factors shaping microclimatic conditions within and around PV solar facilities are highly interdependent. Our review found inconsistent methods and sensor placements across studies, which limits both the ability to capture these dynamic interactions and the capacity for cross‐study comparisons. For example, in 41.2% (14/34) of studies that measured soil temperature, 92.9% (13/14) reported cooling beneath PV panels, often by up to 10 °C, particularly in arid and semi‐arid regions (Broadbent *et al*., 2019; Zheng *et al*., 2023). This co‐occurred with a reduction of soil evaporation, which likely contributed to increases in below‐panel soil moisture observed in studies set in desert environments, especially where runoff from panels accumulated at the dripline (Sturchio *et al*., 2024b). Among studies measuring wind speed, 5 of 7 (71.4%) reported reductions of 30–70% (Zheng *et al*., 2023); however, most microclimate studies did not measure wind speed, which interacts with temperature and evaporation rates. These relationships (e.g. temperature, moisture, and wind) were not examined across studies, confounding the effects of altered temperature and humidity that were observed above and below PV panels. Reductions in solar radiation beneath PV panels can lower surface air and soil surface temperature, which interacts with temperature to control evaporation rates, affecting the intensity and duration of relative humidity and modulating convective heat loss through changes in airflow (Fig. 3). Because these interactions are undoubtedly different than those in natural settings adjacent to a solar facility, characterising whether a PV solar facility creates a cooling or heating effect must include consideration of how abiotic variables interact in context‐dependent ways.

An example of the interplay between these variables is the PVHI effect, in which large‐scale solar installations can create localised above‐ground heat islands, similar to the urban heat island effect (Millstein & Menon, 2011; Barron‐Gafford *et al*., 2016). This effect results from light absorption and re‐radiation of heat during the day, reduced longwave radiation loss at night, and altered land–atmosphere energy fluxes associated with PV infrastructure (Fthenakis & Yu, 2013; Broadbent *et al*., 2019). Traditional monofacial panels have low albedo compared to many natural surfaces, absorbing incoming radiation and converting it to heat rather than reflecting it, which amplifies daytime heat buildup. Emerging bifacial panel technology, designed to capture both direct sunlight and radiation reflected from the ground surface, may further influence ground‐level albedo dynamics and microclimate conditions, although empirical studies examining their ecological effects remain limited. Reduced airflow beneath panels further limits convective heat transfer, potentially amplifying heat accumulation during the day and retention at night. However, Guoqing *et al*. (2021) found that in arid environments, land surface temperatures exhibited a cooling effect, which they attributed to increased effective albedo and lateral dispersion of cooler air from beneath panels. These contrasting observations likely reflect differences in sensor placement relative to panel height, underscoring the need for methodological consistency when comparing across studies.

Although the PVHI effect has been observed in some desert settings, its magnitude and ecological significance vary considerably depending on local climate and facility conditions (Barron‐Gafford *et al*., 2016). In arid regions characterised by high solar irradiance, minimal vegetative cover, low humidity, and soils with low heat capacity, conditions favour rapid daytime heating, nighttime heat retention, and limited evaporative cooling (Fthenakis & Yu, 2013; Barron‐Gafford *et al*., 2016; Broadbent *et al*., 2019; but see Wu *et al*., 2024). By contrast, studies in China's Qinghai Desert (Yue *et al*., 2021; Wu *et al*., 2024) found a less pronounced PVHI effect, possibly due to increased soil moisture from seasonal rainfall promoting cooling. Similarly, studies in less arid environments (e.g. Vervloesem *et al*., 2022) reported weaker PVHI effects, likely because higher baseline humidity and vegetative cover enhanced evapotranspiration and reduced soil temperature variation. This variation among biomes highlights the context dependence of PVHI on baseline climatic conditions. Additionally, inconsistencies in sensor height, depth, and positioning across studies must be considered when interpreting and comparing results.

While some areas of PV solar facilities might exhibit elevated air temperatures, the shaded areas beneath PV panels may exhibit lower soil surface and subsurface temperatures. This shading effect might reduce soil surface temperatures by 5–11 °C during peak solar hours, dampening the extreme heat loads that characterise many arid environments (Tanner *et al*., 2020, 2021; Yue *et al*., 2021). For example, in a desert ecosystem in the USA, daytime sub‐panel air temperature was decreased by an average of 1.2 ± 0.3 °C (mean ± SD) (Barron‐Gafford *et al*., 2019) while air temperatures in Malaysia showed no significant change despite documented reductions in wind speed and soil temperature (Noor & Reeza, 2022). In addition to moderating surface heating, shading alters soil moisture retention, reducing evaporative water loss and potentially creating more stable microclimates that support plant establishment and wildlife burrowing behaviour (Wu *et al*., 2024). Furthermore, PV infrastructure likely redistributes soil moisture and mitigates extreme temperature fluctuations, particularly in arid environments (e.g. Guoqing *et al*., 2021; Wu *et al*., 2022), where shading limits evaporative water loss and allows for enhanced soil water retention (Choi *et al*., 2023). However, the functional significance of these cooler microhabitats remains uncertain, as the thermal properties of PV‐induced shade may differ from those of natural refugia and warrant further research. The cooling effects likely also act as thermal refugia for native species in a warming world, but no empirical study has investigated this aspect.

### (3) The role of convection and insulation

In addition to potential localised heating and cooling effects, alterations to the convection dynamics above and below solar panels are crucial in shaping the local microclimates in a PV facility. Depending on their orientation, tilt, solar tracking, array density, and height above the ground, PV panels block or otherwise alter air movement both at the soil surface and above ground, such that heat dissipation *via* convection is reduced (Guoqing *et al*., 2021; Xu *et al*., 2024). Our review found that in studies measuring wind speed, PV arrays reduce airflow, with wind speeds under panels 30–56% lower compared to open or control areas (e.g. Noor & Reeza, 2022; Zheng *et al*., 2023). This suppression of airflow, particularly in fixed‐tilt arrays with low clearance, may limit convective heat loss and contribute to aboveground heat accumulation, a process associated with PVHI effects (Broadbent *et al*., 2019). This airflow reduction might lead to localised heat retention during the day and can delay nocturnal cooling, particularly in low‐clearance installations. Consequently, convective limitations could compound radiative heat gain and contribute to observed PVHI effects in and around solar facilities. Furthermore, the increased insulation and lack of air mixing below panels may create a less variable and more stable temperature regime. In cases where temperatures under panels are shifted and remain stable for extended periods, the increased effective albedo, combined with the lateral transfer of cool air from under the solar panel, may paradoxically cause localised cooling effects. Thus, it is crucial to focus more research investigating species‐specific responses (e.g. physiologically and behaviourally) to these modified microclimates beneath PV solar arrays.

Microclimates are a complex interplay of multiple environmental variables. What remains unknown is whether these PV solar‐induced microclimates can provide suitable conditions for plants and wildlife by providing a complex thermal–hydrological refugium similar to plants. For example, vegetation of similar height as typical PV solar panels (e.g. 0.5–1 m and less than 2.5 m at the top edge; Williams *et al*., 2023) contributes humidity *via* evapotranspiration and structural complexity. Thus, PV panels likely create different microclimatic conditions than native, lower‐growing vegetation and elicit varying physiological and behavioural responses. It is unclear if or how PV panels may be able to support the same ecological functions as natural shade (Stark *et al*., 2023). Additionally, agrivoltaic systems with panels elevated above typical heights, although relatively uncommon, can create microclimate conditions characterised by cooler temperatures and increased airflow (Williams *et al*., 2023). While the shade provided may moderate thermal extremes, the lack of multi‐dimensional features and complex integration of microclimate variables that plant canopies provide may disrupt the ability of PV panels to support the same ecological functions as natural shade. Depending on the height, orientation, tilt, and solar‐tracking capacity of the panels, convective conditions under the panels may either shift or remain constant for longer durations. These conditions may be thermally favourable for some organisms but unsuitable for others, particularly species that rely on short‐term thermal variability or complex vertical structure for predator avoidance, foraging, or physiological regulation.

### (4) Connecting microclimate shifts to wildlife responses

A key finding of this review was that microclimatic effects vary not only with geography but also with panel height and racking system designs (e.g. fixed *versus* single axis). For example, soil temperatures were consistently lower under panels in most studies, while soil moisture increased in all desert‐region studies that measured it. These cooler, moist conditions may be beneficial to burrowing species or extend the active periods of ectotherms in otherwise inhospitable conditions (e.g. Yue *et al*., 2021; Wu *et al*., 2024). Conversely, above‐panel and inter‐row air temperatures were elevated up to 4 °C, potentially intensifying heat stress for aerial species such as birds and bats (Barron‐Gafford *et al*., 2016; Broadbent *et al*., 2019). However, no studies recorded data more than 2.7 m above the panel surface, and we do not know the spatial (i.e. vertical gradient) or the temporal extent (i.e. dissipation times) of heating or cooling effects above panels. Collectively, these findings indicate that PV solar facilities create spatially heterogeneous microclimates that may modify thermal habitats for species.

Relatively few studies provide direct evidence linking abiotic alterations from PV solar facilities to observed ecological outcomes (but see Suuronen *et al*., 2017; Young *et al*., 2024; Carvalho *et al*., 2025; Sturchio & Knapp, 2025). Nevertheless, there is an increasing acknowledgement that the thermo‐spatial alterations to natural landscapes created by PV solar facilities could potentially exacerbate or mitigate the biophysical challenges species face (Fleckenstein *et al*., 2025) and could have effects on species with environmental sex determination (Schwanz & Georges, 2021). However, such microhabitats may provide localised thermal refugia, a potentially valuable feature in warming climates, particularly for ectotherms whose performance and survival hinge on fine‐scale thermal conditions (Kearney, Shine & Porter, 2009; Huey *et al*., 2012; Sears *et al*., 2016; Doucette *et al*., 2023). Shaded areas under panels may serve as thermal refugia during extreme heat, potentially extending activity windows and reducing evaporative water loss, as well as extending nighttime activity, due to heat retention under solar panels (Tanner *et al*., 2020; Yue *et al*., 2021). However, the quality of artificial refugia may affect species differently, depending on their tolerance of altered shade quality. Yet, the direction and magnitude of all these effects remain context dependent, varying with local climate, panel geometry, species traits, and landscape configuration.

Studies reporting the effects of solar‐induced microclimate on species, both on and off a PV solar facility, are limited. Studies have reported differences in invertebrate and vegetation community composition (Suuronen *et al*., 2017; Graham *et al*., 2021), increases and decreases in reproductive measures in plant species (Hernandez *et al*., 2020; Tanner *et al*., 2021), and increases in primary productivity (Sturchio *et al*., 2024a) due to microclimate shifts within PV solar facilities. Additionally, Enochs *et al*. (2025) reported increased temperatures in bird nests during the non‐breeding season that were constructed directly underneath panels on the metal infrastructure; however, the effect of temperature increases on the reproduction of most vertebrate groups remains unknown. Moreover, there is evidence that PV solar facilities can create stable microclimates that support plant establishment and wildlife burrowing behaviour (Wu *et al*., 2024), which may mitigate extreme temperature fluctuations, particularly in arid environments (Choi *et al*., 2023).

PV solar facilities may produce heating or cooling effects in adjacent areas, and the ability to detect these effects can depend on the height of the sensors. In general, a PVHI effect was reported more often when temperature measurements were taken at or above 1.5 m off the ground (Barron‐Gafford *et al*., 2016; Broadbent *et al*., 2019). Elevated air temperatures above panels may exacerbate thermal stress seen in above‐ground environments, and aerial and above‐ground animals like tree‐nesting birds, flying insects, bats, and other aerial organisms might experience elevated heat stress near PV solar facilities, similar to climate warming studies (Hernandez *et al*., 2014; Armstrong *et al*., 2014). These increased air temperatures can be detected over 300 m outside the PV solar facility with consequences to plants (e.g. water and heat stress; Devitt *et al*., 2022), and potentially non‐volant wildlife (Lovich & Ennen, 2011; DeGregorio *et al*., 2015). Based on our review, no studies have explicitly linked PVHI effects or elevated air temperatures associated with solar installations to adverse effects on volant or non‐volant species, thus highlighting the need for focus on species‐specific responses.

## V. CONCLUSIONS

(1) Our review revealed substantial inconsistencies in sensor placement and reporting across studies, including inconsistent reporting of air and soil temperature heights and depths, as well as treatment locations (e.g. on‐ *versus* offsite controls).
(2) The variability in recorded variables, sensor locations, and measurement heights and depths is likely attributable to differing research questions, study organisms or groups, and budgetary constraints. Such variability complicates cross‐site comparisons and undermines efforts to develop predictive, species‐specific habitat models.
(3) Few studies have reported both surface and subsurface data concurrently, limiting insights into the vertical stratification of thermal environments, which is critical for understanding the ecology of ground‐dwelling *versus* aerial fauna.
(4) The spatial heterogeneity of microclimates created by PV solar facilities, combined with their geographic location, installation, and maintenance methods, presents both challenges and opportunities for optimising outcomes in conservoltaic (biodiversity + PV), ecovoltaic (ecology + PV), and agrivoltaic (agriculture + PV) systems (Nordberg, Caley & Schwarzkopf, 2021; Nordberg & Schwarzkopf, 2023; Sturchio & Knapp, 2023; Knapp & Sturchio, 2024; Omer *et al*., 2025).
(5) Establishing standardised measures of environmental conditions under and around solar panels, including air and soil temperature, humidity, soil moisture, PAR, and airflow, would significantly enhance the value of future microclimate studies (Bacon *et al*., 2024). To facilitate cross‐study comparisons and meta‐analyses, we recommend that future research consistently report key PV facility attributes, including: (*i*) panel mounting system (fixed‐tilt *versus* single‐axis tracking) and design; (*ii*) panel height above ground (minimum and maximum); (*iii*) inter‐row spacing; (*iv*) facility capacity (MW) and land size (ha); (*v*) ecosystem or land‐use type; (*vi*) geographic coordinates and climate zone; (*vii*) groundcover management practices (e.g. grazing, mowing, native vegetation); and (*viii*) sensor placement details, including height, depth, and position relative to panels (e.g. beneath, inter‐row, drip line, or control).
(6) Adoption of standardised approaches and protocols will facilitate cross‐site comparisons and meta‐analyses, enabling the development and calibration of microhabitat suitability models for various species (Martin *et al*., 2012; Kearney & Porter, 2020).
(7) Standardised microclimate data would allow researchers and land managers to predict which areas within solar installations may serve as refuges or stress zones for different taxa, thereby informing the design of solar facilities and guiding targeted habitat enhancement strategies (e.g. planting native wildflowers, installing artificial logs or rock piles, creating shaded patches, or establishing shallow water features).
(8) Ultimately, integrating comprehensive microclimate data with ecological modelling will be crucial for leveraging the potential of solar installations as innovative ecosystems that support biodiversity (Nordberg *et al*., 2021; Boscarino‐Gaetano, Vernes & Nordberg, 2024) and/or agricultural practices (Ali, 2024), thereby reducing land‐use conflicts and maximising co‐benefits.

## AUTHOR CONTRIBUTIONS

T.A.: Writing – Original draft preparation, Methodology, Visualisations, and Data curation; C.L.L.: Conceptualisation, Writing, Editing, and Reviewing; J.R.E.: Conceptualisation, Reviewing, and Editing; K.H.W.: Conceptualisation, Visualisations, Writing, and Reviewing; E.J.N.: Conceptualisation, Methodology, Writing, Editing, Reviewing, and Supervision.

## CONFLICT OF INTEREST STATEMENT

The authors declare that there are no competing interests.

## Supporting information


**Table S1.** Characteristics and environmental measurements from 34 studies derived from 33 publications included in the systematic review.

## Data Availability

No new data were generated from this literature review; all data extracted from the literature cited throughout this manuscript are provided in Table S1.
